# Impact of Temporomandibular Joint Complaints on Tinnitus-Related Distress

**DOI:** 10.3389/fnins.2019.00879

**Published:** 2019-08-22

**Authors:** Niklas K. Edvall, Edis Gunan, Eleni Genitsaridi, Andra Lazar, Golbarg Mehraei, Mattias Billing, Marie Tullberg, Jan Bulla, Jonathon Whitton, Barbara Canlon, Deborah A. Hall, Christopher R. Cederroth

**Affiliations:** ^1^Laboratory of Experimental Audiology, Department of Physiology and Pharmacology, Karolinska Institutet, Stockholm, Sweden; ^2^National Institute of Health Research, Nottingham Biomedical Research Centre, Ropewalk House, Nottingham, United Kingdom; ^3^Hearing Sciences, Division of Clinical Neuroscience, School of Medicine, University of Nottingham, Nottingham, United Kingdom; ^4^Hörsel och Balansmottagningen, Karolinska Universitetssjukhuset, Stockholm, Sweden; ^5^Decibel Therapeutics, Inc., Boston, MA, United States; ^6^Brahekliniken, Stockholm, Sweden; ^7^Department of Mathematics, University of Bergen, Bergen, Norway; ^8^Department of Psychiatry and Psychotherapy, University of Regensburg, Regensburg, Germany; ^9^University of Nottingham Malaysia, Semenyih, Malaysia

**Keywords:** tinnitus, TMJ, stress, distress, somato sensory

## Abstract

There is increasing evidence of associations between the presence of temporomandibular joint (TMJ) disorders and tinnitus. It has been recently proposed that tinnitus patients with TMJ complaints could constitute a subtype, meaning a subgroup of tinnitus patients responsive to specific treatments. Tinnitus patients with TMJ complaints are often young women with somatosensory features of their tinnitus. Here, we investigate the socio-economic factors, phenotypic characteristics and psychological variables of tinnitus subjects from the Swedish Tinnitus Outreach Project, with (*n* = 486) or without (*n* = 1,996) TMJ complaints. The prevalence of TMJ complaints was greater in tinnitus subjects with severe tinnitus (36%) when compared to those with any tinnitus (19%), strongly indicating the contribution of TMJ problems to the severity of tinnitus. Comparing subgroups with or without TMJ complaints in the whole sample, differences were found regarding a large number of socioeconomic, phenotypic, and psychological characteristics. Subjects with TMJ complaints were more often women, more often reported stress as the cause of tinnitus, were more severely affected by tinnitus, scored worse in measures of psychological well-being and life quality, and were more often affected by problems tolerating sounds, headache, vertigo/dizziness, and neck pain. In addition, they more often reported pulsating and tonal tinnitus, somatic modulation of tinnitus, and aggravation of tinnitus by loud sounds and stress. When focusing the analysis in subjects with tinnitus as a big problem using the Tinnitus Functional Index cut-off ≥ 48, or with severe tinnitus according to the Tinnitus Handicap Inventory cut-off ≥ 58, the impact of somatosensory modulations and stress on tinnitus were greater in subjects with TMJ complaints in comparison to those without. In light of these results, we hypothesize that stress could contribute to the co-occurrence of TMJ problems and tinnitus and also to the development of severe tinnitus. Our study supports the need of involving dental care and stress management in the holistic treatment of patients with severe tinnitus.

## Introduction

Subjective tinnitus, ringing in the ears, is a prevalent phantom sound perception (prevalence ranging from 5 to 43%) that in many cases can be severe to the point of seeking medical care (McCormack et al., 2016). Existing treatments are unsatisfactory and are of limited efficacy (Cederroth et al., 2013, 2018; Langguth et al., 2018). *Severe* tinnitus, defined as clinically significant tinnitus, or according to the Tinnitus Handicap Inventory (THI), has been shown to be strongly associated with anxiety and depression (Dobie, 2003; Bartels et al., 2008; Kehrle et al., 2016). Studies have also shown an important contribution of stress as a psychiatric co-morbidity (Hebert et al., 2012; Schlee et al., 2017a) with an overall damaging impact on life quality and subsequent healthcare costs (Stockdale et al., 2017). Sexual dimorphism on the psychological impact of tinnitus has also been revealed, whereby tinnitus is associated with an increased risk in suicide attempts only in women (Lugo et al., 2019).

The mechanisms by which tinnitus occurs are still poorly understood. The current pathophysiological models stipulate that tinnitus emerges as a failure to adapt to missing sensory information originating from the ear (Shore et al., 2016). This results in a compensatory mechanism in the brain translating into a greater neuronal activity (central gain) along the auditory pathway (Auerbach et al., 2014). Limbic structures (e.g., amygdala), that are involved in emotional processing, have been shown to be connected to the auditory pathway and it has been proposed that these contribute to tinnitus (Rauschecker et al., 2010). Interestingly, there is a striking resemblance between the pathways that are involved in tinnitus and chronic pain, converging toward frontostriatal circuits including the ventromedial prefrontal cortex (vmPFC) and the nucleus accumbens (NAc) (Rauschecker et al., 2015).

Recent evidence point toward a dysregulation of multisensory circuits in the generation of tinnitus (Haider et al., 2017). Somatosensory modulations of tinnitus are thought to emerge from abnormal plasticity in somatic-auditory interactions. The dorsal cochlear nucleus is a well-established site of sensory integration (Roberts et al., 2010; Wu et al., 2015). Fusiform cells are at the crossroad of auditory and somatic inputs; on their basal dendrites they receive glutamatergic input from auditory nerve fibers, and on their apical dendrites they receive input from parallel fibers. These inputs also impact on inhibitory interneurons such as cartwheel cells and vertical cells that directly synapse on fusiform cells to provide feed-forward inhibition. Supporting the importance of this integration node, bimodal stimulation of fusiform cells (auditory and somatosensory) has been proposed as an approach to reduce tinnitus in animals and humans (Marks et al., 2018).

It is now agreed that tinnitus is a heterogeneous condition that cannot be considered a single entity (Cederroth et al., 2019a). This may explain why existing interventions are not successful for all patients, and thus identifying “subtypes” is needed to tailor treatments according to a patient profile and improve therapeutic outcome. Common factors related to tinnitus include hearing impairment, head/neck injury, ear infections, depression, and non-steroidal anti-inflammatory drugs (NSAIDs) (Nondahl et al., 2011), but less understood are the contributions of dental problems to tinnitus severity. The cochlea is located just next to the temporomandibular joint, where nerve connections have been mapped from the joint region toward the cochlea. Temporomandibular joint (TMJ) disorders are frequently observed in dental care with a 18–27% prevalence in the population (Deng et al., 1995; Al-Khotani et al., 2016). These can be caused by trauma, an injury or dislocation in the disk of the temporomandibular joint, excessive stress on the jaw muscles (e.g., bruxism), arthritis or malocclusion (Sharma et al., 2011; Murphy et al., 2013; Chisnoiu et al., 2015). Symptoms include pain in the jaw and/or face, difficulties opening the jaw and chewing, and popping sounds when opening the mouth (e.g., when talking or chewing) (Bernhardt et al., 2011). Interestingly, prevalence of tinnitus in patients with TMJ disorders reaches 60% in comparison to 15–30% in patients with no TMJ disorder (Chole and Parker, 1992; Tuz et al., 2003). The association between tinnitus and TMJ disorders was confirmed by two recent systematic reviews that reported a significant association between the two conditions in the majority of the identified studies (Bousema et al., 2018; Mottaghi et al., 2019). Both reviews assessed the quality of the included studies. Quality ranged from low to high indicating some risk of bias in reported findings. Bousema et al. (2018) evaluated 22 studies which investigated the presence of cervical spine disorders (CSDs) or TMJ disorders in patients with and without tinnitus, or the presence of tinnitus in patients with and without TMJ disorders. In contrast, Mottaghi et al. (2019) evaluated findings from just five studies comparing patients with and without TMJ disorders.

Using data from the Tinnitus Research Initiative (TRI) database, Vielsmeier et al. (2012) performed a first in-depth analysis of the characteristics of tinnitus patients with or without self-reported TMJ complaints and found that a larger proportion of those with TMJ complaints were female, younger, and could modulate tinnitus by head/jaw movements or had their tinnitus reduced in the presence of sound (Vielsmeier et al., 2012). However, the study consisted of clinical tinnitus patients only and does not allow for a global picture of the relationship of TMJ complaints with tinnitus in the general population. Furthermore, the questionnaires used in the Vielsmeier et al. (2012) study did not include measures of socioeconomic status, stress, anxiety and emotional reactivity to tinnitus, as measured by the Fear of Tinnitus Questionnaire (FTQ) and the Tinnitus Catastrophising Scale (TCS) (Cima et al., 2012). Here, we will examine the relationship between self-reported TMJ problems with tinnitus using data from the Swedish Tinnitus Outreach Project and investigate a broader range of variables that may differ in tinnitus subjects with or without TMJ complaints.

## Materials and Methods

### Sample

Participants were invited to join the Swedish Tinnitus Outreach Project via social media channels and through partnerships with local cohorts, including LifeGene (Almqvist et al., 2011). All participants above 18 years of age were eligible. Voluntary registration was done on a website from STOP^1^. After providing informed consent for having their data stored in a database and analyzed, participants were invited to an online survey. Between November 2015 and January 2018, 5,593 participants answered the online questionnaires. The present study focused on tinnitus subjects with or without TMJ complaints and their sociodemographic data are reported in Table 1. The project has been approved by the Regional Ethics Review Board in Stockholm (2015/2129-31/1).

**TABLE 1:** Sociodemographic characteristics for subjects with tinnitus with or without TMJ complaints.

	**Any tinnitus**		**TFI ≥ 48**		**THI ≥ 58**	
			
	**TMJ (*n* = 486)** **No (%)**	**No TMJ (*n* = 1996) No (%)**	**TMJ (*n* = 97)** **No (%)**	**No TMJ (*n* = 224)** **No (%)**	**TMJ (*n* = 69)** **No (%)**	**No TMJ (*n* = 122)** **No (%)**
**Sex**	χ^2^(1) = 75.22, *p* < 0.0001 (0.0001)		χ^2^(1) = 6.22, *p* = 0.0127 (0.0762)		χ^2^(1) = 1.92, *p* = 0.166 (0.249)	

Male	161 (33.1)	1099 (55.1)	39 (40.2)	124 (55.4)	29 (42)	64 (52.5)
Female	325 (66.9)	897 (44.9)	58 (59.8)	100 (44.6)	40 (58)	58 (47.5)

**Age group**	χ^2^(7) = 24.18, *p* = 0.0011 (0.0017)		χ^2^(7) = 13.99, *p* = 0.0513 (0.077)		χ^2^(7) = 12.58, *p* = 0.0831 (0.1662)	

<24	13 (2.7)	43 (2.2)	3 (3.1)	4 (1.8)	4 (5.9)	3 (2.5)
25–34	115 (23.8)	371 (18.6)	17 (17.7)	33 (14.8)	14 (20.6)	25 (20.7)
35–44	110 (22.8)	445 (22.3)	25 (26)	39 (17.5)	19 (27.9)	17 (14.1)
45–54	138 (28.6)	525 (26.4)	28 (29.2)	64 (28.7)	17 (25)	29 (24)
55–64	67 (13.9)	285 (14.3)	17 (17.7)	36 (16.1)	10 (14.7)	24 (19.8)
65–74	35 (7.3)	272 (13.7)	5 (5.2)	31 (13.9)	4 (5.9)	16 (13.2)
75–84	4 (0.8)	48 (2.4)	0 (0)	14 (6.3)	0 (0)	6 (5)
>85	1 (0.2)	3 (0.2)	1 (1)	2 (0.9)	0 (0)	1 (0.8)

**Marital status**	χ^2^(4) = 10.18, *p* = 0.0375 (0.045)		χ^2^(4) = 1.93, *p* = 0.7485 (0.8148)		χ^2^(4) = 4.12, *p* = 0.3905 (0.3905)	

Married	183 (37.7)	889 (44.5)	34 (35.1)	90 (40.2)	24 (34.8)	48 (39.3)
Living with partner	161 (33.1)	594 (29.8)	35 (36.1)	68 (30.4)	26 (37.7)	38 (31.2)
Single	102 (21)	333 (16.7)	20 (20.6)	42 (18.8)	16 (23.2)	23 (18.9)
Widow/widower	6 (1.2)	25 (1.3)	1 (1)	5 (2.2)	0 (0)	4 (3.3)
Divorced	34 (7)	155 (7.8)	7 (7.2)	19 (8.5)	3 (4.4)	9 (7.4)

**Gross income**	χ^2^(3) = 27.49, *p* < 0.0001 (0.0001)		χ^2^(3) = 0.94, *p* = 0.8148 (0.8148)		χ^2^(3) = 3.95, *p* = 0.2674 (0.3209)	

0–200,000 SEK	70 (14.4)	232 (11.6)	17 (17.5)	44 (19.6)	13 (18.8)	27 (22.1)
200,001–450,000 SEK	262 (53.9)	944 (47.3)	54 (55.7)	113 (50.5)	44 (63.8)	61 (50)
450,001 SEK or more	122 (25.1)	739 (37)	19 (19.6)	52 (23.2)	9 (13)	23 (18.9)
Don’t know/don’t want to disclose	32 (6.6)	81 (4.1)	7 (7.2)	15 (6.7)	3 (4.4)	11 (9)

**Education level**	χ^2^(3) = 2.39, *p* = 0.495 (0.495)		χ^2^(3) = 7.88, *p* = 0.0486 (0.077)		χ^2^(3) = 7.67, *p* = 0.0535 (0.1605)	

Middle School	17 (3.5)	53 (2.7)	4 (4.1)	17 (7.6)	3 (4.4)	10 (8.2)
High School	108 (22.2)	403 (20.2)	35 (36.1)	60 (26.8)	26 (37.7)	32 (26.2)
University	314 (64.6)	1353 (67.8)	38 (39.2)	117 (52.2)	26 (37.7)	66 (54.1)
Other	47 (9.7)	187 (9.4)	20 (20.6)	30 (13.4)	14 (20.3)	14 (11.5)

**Employment**	χ^2^(10) = 48.23, *p* < 0.0001 (0.0001)		χ^2^(8) = 17.18, *p* = 0.0283 (0.077)		χ^2^(7) = 14.21, *p* = 0.0476 (0.1605)	

Don’t know	0 (0)	1 (0.1)	0 (0)	0 (0)	0 (0)	0 (0)
Employed	313 (64.4)	1219 (61.1)	56 (57.7)	104 (46.4)	44 (63.8)	54 (44.3)
Unemployed	8 (1.7)	22 (1.1)	3 (3.1)	6 (2.7)	1 (1.5)	5 (4.1)
Running my own business/working as a partner in a company	48 (9.9)	259 (13)	6 (6.2)	29 (13)	4 (5.8)	13 (10.7)
Retired	37 (7.6)	289 (14.5)	8 (8.3)	44 (19.6)	4 (5.8)	21 (17.2)
Sick leave (for more than 2 month) or disability pension due to illness or disability	35 (7.2)	63 (3.2)	13 (13.4)	27 (12.1)	8 (11.6)	20 (16.4)
Parental leave (since 2 months or longer)	4 (0.8)	36 (1.8)	1 (1)	4 (1.8)	1 (1.5)	2 (1.6)
Student	24 (4.9)	78 (3.9)	9 (9.3)	6 (2.7)	7 (10.1)	5 (4.1)
Sabbatical	2 (0.4)	3 (0.2)	0 (0)	1 (0.5)	0 (0)	0 (0)
Housewife/-husband	0 (0)	1 (0.1)	0 (0)	0 (0)	0 (0)	0 (0)
Other	15 (3.1)	25 (1.3)	1 (1)	3 (1.3)	0 (0)	2 (1.6)

*Income refers to yearly income in SEK. Pairwise comparisons using Pearson’s Chi-square test are reported. Percentages (%) displayed refer to column percentages. *p*-Values adjusted for multiple comparisons are shown between parenthesis.*

### Questionnaires

The online survey consisted of a combination of standardized questionnaires, which are described in detail elsewhere (Müller et al., 2016). The Tinnitus Sample Case History Questionnaire (TSCHQ) measures phenotypic characteristics that may be associated with tinnitus (Landgrebe et al., 2010), and question #32 “*Do you suffer from temporomandibular disorder?*” was adapted in Swedish to enquire about pain in the jaw muscles or problems in the jaw rather than the diagnosis itself (“*Lider du av smärta i käkmuskeler eller störnig i käkfunktions?*”) (Müller et al., 2016), as laymen may be unfamiliar with the medical terminology. This question lead to an intra-class coherent coefficient of 0.67 (acceptable) in a test–retest (Müller et al., 2016). The THI (Newman et al., 1996) and the Tinnitus Functional Index (TFI) (Meikle et al., 2012) consist of multiple subscales measuring the impact of tinnitus in the different aspects of everyday life and give a global score of tinnitus symptom severity. The Fear of Tinnitus Questionnaire (FTQ) and the Tinnitus Catastrophizing Scale (TCS) measure fear and degree of catastrophic misinterpretations regarding tinnitus (Cima et al., 2011). The Hyperacusis Questionnaire (HQ) evaluates symptomatology related to sensitivity to sound (Khalfa et al., 2002). The Perceived Stress Questionnaire (PSQ) (Levenstein et al., 1993) and the Hospital Anxiety Depression Scales for Anxiety (HADS A) and depression (HADS D) measure stress, anxiety, and depression respectively (Andersson et al., 2003). The World Health Organization’s Quality of Life (WHOQoL)-BREF subscales WHOQol Physical, WHOQol Psychological, WHOQol Social, and WHOQol Environmental, evaluate quality of life domains (The Whoqol Group, 1998). All questionnaires were translated into Swedish and previously administered to tinnitus subjects (Müller et al., 2016). Numerical Rating Scales (NRS) for Loudness, Awareness, and Annoyance were obtained via the TSCHQ (questions 12, 16, and 17). Some of these questionnaires were selected based on their integration in the TRI database^2^.

### Inclusion Criteria

Subjects were included if they answered “Yes, always (all the time)” and “Yes, occasionally (now and then)” (*n* = 2,597) to the question “Do you have tinnitus?” Those who answered “No” or “Don’t know” (*n* = 2,991) and 5 missing entries were excluded from the analysis. The second inclusion criterion was based on whether subjects answered either “Yes” or “No” to the question “Do you suffer from temporomandibular disorder?” Subjects who answered “Don’t know” (*n* = 113) were excluded, and those who answered “Yes” (*n* = 486) and “No” (*n* = 1,996) were included (2 missing entries). Thus, 44% (*n* = 2,482) of the total subjects (*n* = 5,593) were considered in the analysis. Since the classification of subjects is based on self-report, we used the terminology TMJ ‘complaints’ which distinguishes the study group from diagnosed TMJ ‘disorders.’ We also investigated subjects with severe tinnitus. Severe tinnitus was operationally defined in two ways. The first used a revised grading system of the original eight-factor 25 item of the TFI (Fackrell et al., 2017) where a TFI cut-off score ≥ 48 denotes a big problem. The second used a THI cut-off score ≥ 58, since this boundary is used as a criterion for referral to specialty care in the Stockholm County (Idrizbegovic et al., 2011). For some questionnaires, data were missing for 13 participants in the non-TMJ group and 5 participants in the TMJ group.

### Statistical Analysis

Phenotypic characteristics such as tinnitus loudness, pitch, onset, whether tinnitus is pulsating or not, what the tinnitus sounds like, and onset-related events were obtained from the TSCHQ. Additional sociodemographic data (i.e., marital status, income, employment status, and education level) were obtained using questions from Svensson et al. (2013). All statistical analyses were performed in JMP 13 (SAS Institute, Inc.) and R Core Team (2019). For nominal variables, Pearson’s Chi-squared test was used. Homoscedasticity between groups was tested for using the Brown–Forsythe test and showed significant differences between subgroups for multiple variables. Several questionnaire total scores also deviated from a normal distribution. Therefore, the non-parametric Wilcoxon’s test was used for all comparisons to provide easy comparability between different groups for the reader. In order to investigate the potential impact of multiple comparisons on the discovery rates of our tests, we also report *p*-values adjusted by the method of Benjamini and Hochberg. The adjustments were computed for each set of *p*-values resulting from multiple tests carried out.

## Results

Nineteen percent (486/2482) of those with any tinnitus reported TMJ complaints. The socioeconomic characteristics of our study sample with TMJ complaints and tinnitus are listed in Table 1. Subjects with TMJ complaints differed from those without TMJ complaints in sex, age, marital status, gross income, and employment status (*p* = 0.0375 to < 0.0001) and survived correction for multiple comparisons. No difference in education level was found between the two groups. Thirty percent of the sample with tinnitus as a big problem (97/321) (i.e., TFI ≥ 48), reported TMJ complaints. Using the THI cut-off for severity (≥58), 36% (69/191) reported TMJ complaints. In the samples reporting a big problem or with severe tinnitus, no differences in the socioeconomic characteristics were found after correction for multiple comparisons (Table 1). These findings suggest that with increasing severity, socioeconomic differences between groups with or without TMJ complaints no longer exist.

### Greater Tinnitus Symptom Severity in Tinnitus Subjects With TMJ Complaints

Greater tinnitus-related burden was found in tinnitus subjects with TMJ complaints (Table 2). Scores in all the instruments indicated greater impact in tinnitus subjects with TMJ complaints compared to those without TMJ complaints. Differences were statistically significant for all variables (NRS loudness, awareness, annoyance, THI, TFI, FTQ, TCS, HQ, PSQ, HADS A, HADS D, and all quality of life scores; *p* = 0.02 to < 0.0001). When assessing cases with tinnitus as a big problem, fewer questionnaires showed significant differences (FTQ, TCS, HQ, HADS A, HADS D, physical, psychological, and environmental quality of life). In contrast, in subjects with severe tinnitus, no differences were found after correction for multiple comparisons with the exception of the psychological quality of life (*p* = 0.02). Again, with increasing severity, fewer differences were found between subjects with and without TMJ complaints (Table 2).

**TABLE 2:** Questionnaire scores from subjects with tinnitus with or without TMJ complaints.

	**Any tinnitus**		**TFI ≥ 48**		**THI ≥ 58**	
			
	**TMJ (*n* = 486) Mean (SD)**	**No TMJ (*n* = 1996) Mean (SD)**	**TMJ (*n* = 97) Mean (SD)**	**No TMJ (*n* = 224) Mean (SD)**	**TMJ (*n* = 69) Mean (SD)**	**No TMJ (*n* = 122) Mean (SD)**
NRS Lo	44.4 (25.7)	39.8 (25.2)	71.3 (21.3)	71.5 (18.3)	75.3 (17.8)	74.2 (19.7)

	3.57; *p* = 0.0004 (0.0004)		0.29; *p* = 0.7688 (0.8145)		0.21; *p* = 0.8314 (0.8908)	

NRS Aw	36 (32.1)	32.7 (31.1)	72.8 (26.9)	74.9 (24.9)	73.5 (27)	76.8 (24.3)

	2.33; *p* = 0.02 (0.02)		−0.55; *p* = 0.5799 (0.6691)		−0.65; *p* = 0.514 (0.6903)	

NRS An	24.7 (28)	18.7 (24.6)	60.9 (26.6)	60 (27)	63.6 (28.2)	66.7 (26.4)

	5.17; *p* = < 0.0001 (0.0001)		0.23; *p* = 0.8145 (0.8145)		−0.63; *p* = 0.5285 (0.6903)	

THI	28.8 (23.1)	19.6 (19.1)	63.1 (18.3)	58.2 (18.7)	73.3 (12.3)	73.1 (11.5)

	9; *p* < 0.0001 (<0.0001)		2.15; *p* = 0.0312 (0.052)		0.05; *p* = 0.9608 (0.9608)	

TFI	27.8 (23.2)	20.1 (19.8)	65.6 (13.3)	63 (11.9)	66.8 (16.8)	66.6 (14.9)

	6.9; *p* < 0.0001 (<0.0001)		1.52; *p* = 0.1274 (0.1737)		0.28; *p* = 0.7817 (0.8908)	

PSQ	0.4 (0.2)	0.3 (0.2)	0.6 (0.2)	0.5 (0.2)	0.6 (0.2)	0.5 (0.2)

	12.12; *p* < 0.0001 (<0.0001)		4.72; *p* < 0.0001 (0.0001)		2.56; *p* = 0.0104 (0.052)	

HADS_A	8.1 (4.5)	5.6 (3.9)	11.4 (4.6)	8.5 (4.3)	11.8 (4.5)	10 (4.4)

	11.01; *p* < 0.0001 (<0.0001)		4.93; *p* < 0.0001 (0.0001)		2.6; *p* = 0.0092 (0.052)	

HADS_D	4.8 (4)	3.3 (3.1)	8.2 (4.3)	6.3 (3.7)	8.5 (4.5)	7.5 (3.7)

	7.88; *p* < 0.0001 (<0.0001)		4; *p* < 0.0001 (0.0001)		1.64; *p* = 0.1008 (0.3024)	

FTQ	5.6 (3)	4.5 (2.6)	8.6 (2.9)	8 (3)	9.7 (2.7)	9.2 (2.8)

	7.27; *p* < 0.0001 (<0.0001)		1.74; *p* = 0.0825 (0.1238)		1.7; *p* = 0.0895 (0.3024)	

TCS	15.9 (11.5)	11.6 (9.8)	29.2 (9)	26.8 (10.2)	31.4 (7.9)	30.8 (9)

	7.56; *p* < 0.0001 (<0.0001)		2.23; *p* = 0.0258 (0.0484)		0.59; *p* = 0.5522 (0.6903)	

HQ	20.6 (9.1)	15.6 (9)	26.6 (7.6)	23.5 (8.8)	27.4 (7.7)	25.6 (8.9)

	10.68; *p* < 0.0001 (<0.0001)		3.13; *p* = 0.0018 (0.0039)		1.39; *p* = 0.1654 (0.3544)	

QoL Phy	14.4 (3)	16 (2.5)	12 (2.8)	13.5 (2.8)	12.1 (3)	12.6 (2.7)

	−10.68; *p* < 0.0001 (<0.0001)		−4.33; *p* < 0.0001 (0.0001)		−1.27; *p* = 0.2033 (0.3812)	

QoL Psy	13.7 (3)	15.1 (2.5)	11.3 (3.1)	13.2 (2.7)	11 (3.3)	12.4 (2.6)

	−9.25; *p* < 0.0001 (<0.0001)		−5.02; *p* < 0.0001 (0.0001)		−3.21; *p* = 0.0013 (0.0195)	

QoL social	13.6 (3.3)	14.3 (3)	12.6 (3.8)	13.1 (3.2)	12.9 (3.6)	12.4 (3.4)

	−4.76; *p* < 0.0001 (<0.0001)		−1.4; *p* = 0.1603 (0.2004)		0.68; *p* = 0.4986 (0.6903)	

QoL Env	15.5 (2.5)	16.5 (2.1)	13.8 (2.6)	15.1 (2.4)	14.1 (2.8)	14.6 (2.5)

	−8.24; *p* < 0.0001 (<0.0001)		−4.1; *p* < 0.0001 (0.0001)		−1.42; *p* = 0.1552 (0.3544)	

*Values are mean (±SD). Pairwise comparisons using Wilcoxon’s tests are reported below the compared values. *p-*Values adjusted for multiple comparisons are shown between parenthesis. NRS, Numerical Ratins Score; Lo, tinnitus loudness; Aw, awareness; An, annoyance; THI, Tinnitus Handicap Inventory; TFI, Tinnitus Functional Index; FTQ, Fear of Tinnitus Questionnaire; TCS, Tinnitus Catastrophising Scale; HQ, Hyperacusis Questionnaire; PSQ, Perceived Stress Questionnaire; HADS A, Hospital Anxiety Depression Scales for Anxiety; HADS D, Hospital Anxiety Depression Scales for Depression; QoL, quality of life; subscales from the World Health Organization: Phy, physical, Psych, psychological; Soc, social; Env, environmental.*

### Tinnitus Phenotypics in Tinnitus Subjects With and Without TMJ Complaints

We next analyzed the tinnitus phenotypic characteristics of subjects with or without TMJ complaints (Table 3). With any tinnitus, the two groups differed in onset-related events, more frequently reporting stress as the cause of their tinnitus. Differences were also found in pulsatility, location of tinnitus, sound of tinnitus, day to day variations, worsening by loud noise, affected by nap, or bad night sleep (*p* = 0.04 to < 0.0001). No differences were found in tinnitus time of onset, in tinnitus pitch, or in its reduction by music or environmental sounds. Those with TMJ complaints were more likely to contact a clinician due to tinnitus, received a greater number of treatments, and reported greater occurrence in the family (*p* = 0.005 to < 0.0001). Table 4 shows that subjects with TMJ complaints more often experience discomfort in presence of loud noise and problems tolerating sounds (*p* < 0.0001), and reported worse hearing (*p* = 0.04), in spite of similar use of hearing aids (*p* = 0.38). Regarding co-morbidities, subjects with TMJ complaints displayed greater incidence of headache and vertigo, and more often were under psychiatric treatment and had other diagnosed diseases. The factors that were consistently found to differ between subjects with or without TMJ complaints, whether they had any tinnitus, tinnitus as a big problem or severe tinnitus, were modulation by head movement or touch, affected by stress, and neck pain (Tables 3, 4).

**TABLE 3:** Phenotypic characteristics for subjects with tinnitus with or without TMJ complaints.

	**Any tinnitus**		**TFI ≥ 48**		**THI ≥ 58**	
			
	**TMJ (*n* = 486)** **No (%)**	**No TMJ (*n* = 1996) No (%)**	**TMJ (*n* = 97)** **No (%)**	**No TMJ (*n* = 224) No (%)**	**TMJ (*n* = 69)** **No (%)**	**No TMJ (*n* = 122) No (%)**
**Tinnitus onset**	χ^2^(5) = 1.23, *p* = 0.942 (0.942)		χ^2^(5) = 4.36, *p* = 0.499 (0.756)		χ^2^(5) = 3.34, *p* = 0.648 (0.748)	

Don’t know	48 (9.9)	176 (8.8)	2 (2.1)	6 (2.7)	1 (1.5)	3 (2.5)
0 to 6 months	11 (2.3)	39 (2)	4 (4.1)	9 (4)	3 (4.4)	7 (5.7)
6 months to 3 years	70 (14.4)	293 (14.7)	11 (11.3)	39 (17.4)	12 (17.4)	25 (20.5)
3 to 10 years	143 (29.4)	578 (29)	31 (32)	59 (26.3)	21 (30.4)	29 (23.8)
10 to 20 years	140 (28.8)	577 (28.9)	33 (34)	62 (27.7)	22 (31.9)	31 (25.4)
More than 20 years	74 (15.2)	333 (16.7)	16 (16.5)	49 (21.9)	10 (14.5)	27 (22.1)

**Onset-related events**	χ^2^(6) = 50.76, *p* < 0.0001 (0.0001)		χ^2^(6) = 4.64, *p* = 0.591 (0.771)		χ^2^(6) = 6.38, *p* = 0.382 (0.603)	

Loud blast of sound	163 (33.5)	804 (40.3)	27 (27.8)	72 (32.1)	22 (31.9)	33 (27.1)
Stress ^∗∗∗^, #	106 (21.8)	243 (12.2)	18 (18.6)	36 (16.1)	14 (20.3)	21 (17.2)
Change in hearing	26 (5.4)	105 (5.3)	14 (14.4)	23 (10.3)	10 (14.5)	12 (9.8)
Head trauma	5 (1)	15 (0.8)	1 (1)	1 (0.5)	0 (0)	1 (0.8)
Whiplash	9 (1.9)	11 (0.6)	2 (2.1)	2 (0.9)	0 (0)	1 (0.8)
Other	80 (16.5)	265 (13.3)	20 (20.6)	40 (17.9)	15 (21.7)	24 (19.7)
Don’t know	97 (20)	553 (27.7)	15 (15.5)	50 (22.3)	8 (11.6)	30 (24.6)

**Tinnitus occurrence**	χ^2^(1) = 0.18, *p* = 0.671 (0.694)		χ^2^(1) = 3.63, *p* = 0.057 (0.17)		χ^2^(1) = 0, *p* = 0.973 (0.973)	

Occasionally (now and then)	218 (44.9)	874 (43.8)	14 (14.4)	17 (7.6)	5 (7.3)	9 (7.4)
Always (all the time)	268 (55.1)	1122 (56.2)	83 (85.6)	207 (92.4)	64 (92.8)	113 (92.6)

**Time of the day of tinnitus emergence**	χ^2^(6) = 13.02, *p* = 0.043 (0.056)		χ^2^(6) = 3.61, *p* = 0.729 (0.824)		χ^2^(6) = 6.67, *p* = 0.353 (0.588)	

Don’t know	264 (54.3)	1074 (53.8)	42 (43.3)	90 (40.2)	30 (43.5)	34 (27.9)
When awakening	32 (6.6)	120 (6)	14 (14.4)	29 (13)	12 (17.4)	23 (18.9)
In the morning	2 (0.4)	42 (2.1)	1 (1)	9 (4)	1 (1.5)	6 (4.9)
Around noon	22 (4.5)	138 (6.9)	9 (9.3)	26 (11.6)	6 (8.7)	18 (14.8)
In the afternoon	23 (4.7)	96 (4.8)	5 (5.2)	17 (7.6)	5 (7.3)	13 (10.7)
In the evening	58 (11.9)	244 (12.2)	18 (18.6)	34 (15.2)	10 (14.5)	20 (16.4)
Before sleeping	85 (17.5)	282 (14.1)	8 (8.3)	19 (8.5)	5 (7.3)	8 (6.6)

**Perceiving the onset of tinnitus**	χ^2^(2) = 4.03, *p* = 0.133 (0.16)		χ^2^(2) = 0.14, *p* = 0.933 (0.965)		χ^2^(2) = 0.49, *p* = 0.781 (0.837)	

Don’t know	110 (22.6)	540 (27.1)	10 (10.3)	26 (11.6)	6 (8.7)	9 (7.4)
Gradual	222 (45.7)	848 (42.5)	40 (41.2)	93 (41.5)	27 (39.1)	43 (35.3)
Abrupt	154 (31.7)	608 (30.5)	47 (48.5)	105 (46.9)	36 (52.2)	70 (57.4)

**Pulsatility**	χ^2^(3) = 30.48*, p* < 0.0001 (0.0001)		χ^2^(3) = 2.35, *p* = 0.504 (0.756)		χ^2^(3) = 2.4, *p* = 0.494 (0.676)	

Don’t know	59 (12.1)	139 (7)	11 (11.3)	26 (11.6)	4 (5.8)	12 (9.8)
Yes, with heart beat	45 (9.3)	126 (6.3)	10 (10.3)	22 (9.8)	9 (13)	14 (11.5)
Yes, different from heart beat	29 (6)	67 (3.4)	13 (13.4)	18 (8)	11 (15.9)	12 (9.8)
No	353 (72.6)	1664 (83.4)	63 (65)	158 (70.5)	45 (65.2)	84 (68.9)

**Location of tinnitus**	χ^2^(6) = 20.48, *p* = 0.002 (0.004)		χ^2^(6) = 20.15, *p* = 0.003 (0.013)		χ^2^(6) = 10.01*, p* = 0.124 (0.382)	

Right ear	36 (7.4)	159 (8)	9 (9.3)	22 (9.8)	6 (8.7)	10 (8.2)
Left ear	39 (8)	184 (9.2)	9 (9.3)	20 (8.9)	9 (13)	15 (12.3)
Both ears, worse in right	111 (22.8)	319 (16)	26 (26.8)	28 (12.5)	17 (24.6)	19 (15.6)
Both ears, worse in left	76 (15.6)	345 (17.3)	15 (15.5)	53 (23.7)	12 (17.4)	30 (24.6)
Both ears equally	144 (29.6)	657 (32.9)	18 (18.6)	58 (25.9)	11 (15.9)	24 (19.7)
Inside the head	70 (14.4)	317 (15.9)	14 (14.4)	41 (18.3)	9 (13)	23 (18.9)
Elsewhere	10 (2.1)	15 (0.8)	6 (6.2)	2 (0.9)	5 (7.3)	1 (0.8)

**Sound of tinnitus**	χ^2^(9) = 33.6, *p* < 0.0001 (0.0001)		χ^2^(7) = 9.53, *p* = 0.217 (0.406)		χ^2^(7) = 12.39, *p* = 0.088 (0.332)	

Tone	88 (18.4)	442 (22.3)	10 (10.5)	24 (10.9)	10 (14.9)	12 (10)
Noise	36 (7.5)	236 (11.9)	5 (5.3)	27 (12.2)	4 (6)	10 (8.3)
Crickets	18 (3.8)	66 (3.3)	3 (3.2)	5 (2.3)	2 (3)	4 (3.3)
Heartbeat	5 (1)	6 (0.3)	1 (1.1)	1 (0.5)	2 (3)	0 (0)
Beeping	48 (10)	240 (12.1)	10 (10.5)	26 (11.8)	5 (7.5)	14 (11.7)
Morse code	2 (0.4)	1 (0.1)	0 (0)	0 (0)	0 (0)	0 (0)
An alarm	7 (1.5)	12 (0.6)	3 (3.2)	2 (0.9)	2 (3)	2 (1.7)
Other	9 (1.9)	61 (3.1)	1 (1.1)	11 (5)	0 (0)	11 (9.2)
Don’t know	1 (0.2)	1 (0.1)	0 (0)	0 (0)	0 (0)	0 (0)
Complex	265 (55.3)	916 (46.2)	62 (65.3)	125 (56.6)	42 (62.7)	67 (55.8)

**Tinnitus loudness variation from day to day**	χ^2^(5) = 20.74, *p* = 0.001 (0.002)		χ^2^(5) = 12.94, *p* = 0.024 (0.09)		χ^2^(5) = 5.11, *p* = 0.403 (0.604)	

Don’t know	21 (4.3)	142 (7.1)	4 (4.1)	4 (1.8)	2 (2.9)	1 (0.8)
Never	30 (6.2)	165 (8.3)	7 (7.2)	23 (10.3)	4 (5.8)	9 (7.4)
Seldom	55 (11.3)	312 (15.6)	9 (9.3)	40 (17.9)	6 (8.7)	16 (13.1)
Sometimes	185 (38.1)	737 (36.9)	34 (35.1)	96 (42.9)	24 (34.8)	54 (44.3)
Often	139 (28.6)	430 (21.5)	28 (28.9)	43 (19.2)	21 (30.4)	27 (22.1)
Always	56 (11.5)	210 (10.5)	15 (15.5)	18 (8)	12 (17.4)	15 (12.3)

**Pitch of tinnitus**	χ^2^(4) = 3.75*, p* = 0.441 (0.472)		χ^2^(4) = 2.38, *p* = 0.666 (0.824)		χ^2^(4) = 7.16, *p* = 0.128 (0.382)	

Don’t know	23 (4.7)	70 (3.5)	4 (4.1)	5 (2.2)	4 (5.8)	1 (0.8)
Very high frequency	119 (24.5)	482 (24.2)	34 (35.1)	67 (29.9)	28 (40.6)	41 (33.6)
High frequency	206 (42.4)	896 (44.9)	39 (40.2)	102 (45.5)	26 (37.7)	47 (38.5)
Medium frequency	92 (18.9)	394 (19.7)	15 (15.5)	41 (18.3)	8 (11.6)	23 (18.9)
Low frequency	46 (9.5)	154 (7.7)	5 (5.2)	9 (4)	3 (4.4)	10 (8.2)

**Reduction of tinnitus by music or environmental sounds**	χ^2^(2) = 4.25, *p* = 0.12 (0.15)		χ^2^(2) = 1.15*, p* = 0.563 (0.771)		χ^2^(2) = 3.76, *p* = 0.153 (0.382)	

Don’t know	116 (23.9)	511 (25.6)	12 (12.4)	35 (15.6)	7 (10.1)	16 (13.1)
Yes	294 (60.5)	1110 (55.6)	61 (62.9)	127 (56.7)	45 (65.2)	62 (50.8)
No	76 (15.6)	375 (18.8)	24 (24.7)	62 (27.7)	17 (24.6)	44 (36.1)

**Worsening of tinnitus by loud noise**	χ^2^(2) = 18.48, *p* < 0.0001 (0.0001)		χ^2^(2) = 3.32, *p* = 0.19 (0.38)		χ^2^(2) = 1.23, *p* = 0.541 (0.676)	

Don’t know	145 (29.8)	549 (27.5)	26 (26.8)	49 (21.9)	17 (24.6)	31 (25.4)
Yes	263 (54.1)	944 (47.3)	59 (60.8)	129 (57.6)	44 (63.8)	70 (57.4)
No	78 (16.1)	503 (25.2)	12 (12.4)	46 (20.5)	8 (11.6)	21 (17.2)

**Tinnitus affected by head movement or touch**	χ^2^(2) = 122.84, *p* < 0.0001 (0.0001)		χ^2^(2) = 23.09, *p* < 0.0001 (0.0005)		χ^2^(2) = 12.5, *p* = 0.002 (0.019)	

Don’t know	123 (25.3)	376 (18.8)	13 (13.4)	32 (14.3)	7 (10.1)	13 (10.7)
Yes	198 (40.7)	411 (20.6)	55 (56.7)	66 (29.5)	43 (62.3)	45 (36.9)
No	165 (34)	1209 (60.6)	29 (29.9)	126 (56.3)	19 (27.5)	64 (52.5)

**Tinnitus affected by nap**	χ^2^(3) = 9.36, *p* = 0.025 (0.036)		χ^2^(3) = 0.15, *p* = 0.986 (0.986)		χ^2^(3) = 2.24, *p* = 0.525 (0.676)	

Don’t know	290 (59.7)	1126 (56.4)	38 (39.2)	87 (38.8)	24 (34.8)	42 (34.4)
It mainly worsens my tinnitus	13 (2.7)	40 (2)	8 (8.3)	16 (7.1)	8 (11.6)	14 (11.5)
It mainly reduces my tinnitus	133 (27.4)	674 (33.8)	41 (42.3)	98 (43.8)	33 (47.8)	51 (41.8)
It has no effect	50 (10.3)	156 (7.8)	10 (10.3)	23 (10.3)	4 (5.8)	15 (12.3)

**Tinnitus affected by bad nights sleep**	χ^2^(5) = 67.67, *p* < 0.0001 (0.0001)		χ^2^(5) = 8.34, *p* = 0.139 (0.316)		χ^2^(5) = 10.66, *p* = 0.059 (0.293)	

Don’t know	177 (36.4)	762 (38.2)	19 (19.6)	43 (19.2)	9 (13)	20 (16.4)
Never	30 (6.2)	337 (16.9)	3 (3.1)	28 (12.5)	1 (1.5)	13 (10.7)
Seldom	30 (6.2)	158 (7.9)	8 (8.3)	16 (7.1)	6 (8.7)	8 (6.6)
Sometimes	116 (23.9)	440 (22)	25 (25.8)	58 (25.9)	15 (21.7)	31 (25.4)
Often	99 (20.4)	230 (11.5)	27 (27.8)	57 (25.5)	21 (30.4)	36 (29.5)
Always	34 (7)	69 (3.5)	15 (15.5)	22 (9.8)	17 (24.6)	14 (11.5)
Heartbeat	5 (1)	6 (0.3)	1 (1.1)	1 (0.5)	2 (3)	0 (0)

**Tinnitus affected by stress**	χ^2^(3) = 104.08, *p* < 0.0001 (0.0001)		χ^2^(2) = 22.11, *p* < 0.0001 (0.0005)		χ^2^(2) = 14.72, *p* = 0.001 (0.009)	

Don’t know	134 (27.6)	731 (36.6)	15 (15.5)	51 (22.8)	6 (8.7)	24 (19.7)
Yes, it worsens my tinnitus	305 (62.8)	768 (38.5)	78 (80.4)	123 (54.9)	62 (89.9)	80 (65.6)
Yes, it reduces my tinnitus	47 (9.7)	492 (24.7)	4 (4.1)	50 (22.3)	1 (1.5)	18 (14.8)
No, it has no effect	0 (0)	5 (0.3)	0 (0)	0 (0)	0 (0)	0 (0)

**Tinnitus affected by medication**	χ^2^(2) = 3.78, *p* = 0.151 (0.175)		χ^2^(2) = 0.25, *p* = 0.881 (0.944)		χ^2^(2) = 1.38, *p* = 0.502 (0.676)	

Don’t know	356 (73.3)	1467 (73.5)	63 (65)	149 (66.5)	46 (66.7)	73 (59.8)
Yes	18 (3.7)	44 (2.2)	8 (8.3)	15 (6.7)	7 (10.1)	11 (9)
No	112 (23.1)	485 (24.3)	26 (26.8)	60 (26.8)	16 (23.2)	38 (31.2)

**Contacted a clinician due to tinnitus**	χ^2^(2) = 11.42, *p* = 0.003 (0.005)		χ^2^(2) = 0.6, *p* = 0.742 (0.824)		χ^2^(2) = 2.33, *p* = 0.313 (0.588)	

No	282 (58)	1313 (65.8)	23 (23.7)	49 (21.9)	11 (15.9)	18 (14.8)
Yes, because of curiosity	26 (5.4)	107 (5.4)	2 (2.1)	8 (3.6)	0 (0)	4 (3.3)
Yes, because I sought for help	178 (36.6)	576 (28.9)	72 (74.2)	167 (74.6)	58 (84.1)	100 (82)

**Number of tinnitus treatments**	χ^2^(3) = 25.56, *p* < 0.0001 (0.0001)		χ^2^(3) = 4.26, *p* = 0.235 (0.414)		χ^2^(3) = 1.96, *p* = 0.581 (0.697)	

None	395 (81.3)	1776 (89)	55 (56.7)	142 (63.4)	34 (49.3)	66 (54.1)
1	32 (6.6)	102 (5.1)	11 (11.3)	34 (15.2)	9 (13)	19 (15.6)
2–4	43 (8.9)	84 (4.2)	19 (19.6)	29 (13)	17 (24.6)	20 (16.4)
5 or more	16 (3.3)	34 (1.7)	12 (12.4)	19 (8.5)	9 (13)	17 (13.9)

**Tinnitus occurence in family**	χ^2^(1) = 24.97, *p* < 0.0001 (0.0001)		χ^2^(1) = 6.58, *p* = 0.01 (0.044)		χ^2^(1) = 1.68, *p* = 0.195 (0.418)	

No	358 (73.7)	1666 (83.5)	62 (63.9)	174 (77.7)	46 (66.7)	92 (75.4)
Yes	128 (26.3)	330 (16.5)	35 (36.1)	50 (22.3)	23 (33.3)	30 (24.6)

*Pairwise comparisons using Pearson’s Chi-square test are reported. Percentages (%) displayed refer to column percentages. *p-*Values adjusted for multiple comparisons are shown between parenthesis and included variables from Table 4.*

**TABLE 4:** Comorbidities in subjects with tinnitus with or without TMJ complaints.

	**Any tinnitus**		**TFI ≥ 48**		**THI ≥ 58**	
			
	**TMJ (*n* = 486)** **No (%)**	**No TMJ (*n* = 1996) No (%)**	**TMJ (*n* = 97)** **No (%)**	**No TMJ (*n* = 224) No (%)**	**TMJ (*n* = 69)** **No (%)**	**No TMJ (*n* = 122) No (%)**
**Hearing problem**	χ^2^(2) = 7.25, *p* = 0.027 (0.036)		χ^2^(2) = 4.17, *p* = 0.124 (0.311)		χ^2^(2) = 3.46, *p* = 0.177 (0.409)	

Don’t know	100 (20.6)	310 (15.5)	14 (14.4)	23 (10.3)	6 (8.7)	11 (9)
Yes	230 (47.3)	996 (49.9)	59 (60.8)	162 (72.3)	38 (55.1)	82 (67.2)
No	156 (32.1)	690 (34.6)	24 (24.7)	39 (17.4)	25 (36.2)	29 (23.8)

**Hearing aids**	χ^2^(3) = 3.37, *p* = 0.338 (0.376)		χ^2^(3) = 2.03, *p* = 0.567 (0.771)		χ^2^(3) = 1.53, *p* = 0.676 (0.751)	

Yes, on both ears	38 (7.8)	117 (5.9)	23 (23.7)	41 (18.3)	14 (20.3)	20 (16.4)
Yes, on the right ear	6 (1.2)	17 (0.9)	4 (4.1)	6 (2.7)	3 (4.4)	4 (3.3)
Yes, on the left ear	5 (1)	25 (1.3)	2 (2.1)	7 (3.1)	1 (1.5)	5 (4.1)
No	437 (89.9)	1837 (92)	68 (70.1)	170 (75.9)	51 (73.9)	93 (76.2)

**Problems tolerating sounds**	χ^2^(4) = 50.16, *p* < 0.0001 (0.0001)		χ^2^(4) = 2.08, *p* = 0.721 (0.824)		χ^2^(3) = 0.34, *p* = 0.952 (0.973)	

Never	7 (1.4)	99 (5)	0 (0)	1 (0.5)	0 (0)	0 (0)
Rarely	50 (10.3)	350 (17.5)	5 (5.2)	13 (5.8)	4 (5.8)	6 (4.9)
Sometimes	176 (36.2)	800 (40.1)	21 (21.7)	62 (27.7)	14 (20.3)	29 (23.8)
Usually	153 (31.5)	498 (25)	36 (37.1)	79 (35.3)	24 (34.8)	41 (33.6)
Always	100 (20.6)	249 (12.5)	35 (36.1)	69 (30.8)	27 (39.1)	46 (37.7)

**Sounds cause pain or physical discomfort**	χ^2^(2) = 58.18, *p* < 0.0001 (0.0001)		χ^2^(2) = 5.5, *p* = 0.064 (0.175)		χ^2^(2) = 5.27, *p* = 0.072 (0.308)	

Don’t know	34 (7)	111 (5.6)	6 (6.2)	13 (5.8)	3 (4.4)	7 (5.7)
Yes	326 (67.1)	989 (49.6)	77 (79.4)	152 (67.9)	59 (85.5)	87 (71.3)
No	126 (25.9)	896 (44.9)	14 (14.4)	59 (26.3)	7 (10.1)	28 (23)

**Headache**	χ^2^(2) = 164.05, *p* < 0.0001 (0.0001)		χ^2^(2) = 15.93, *p* = 0.0003 (0.002)		χ^2^(2) = 8.99, *p* = 0.011 (0.084)	

Don’t know	10 (2.1)	34 (1.7)	2 (2.1)	4 (1.8)	2 (2.9)	4 (3.3)
Yes	231 (47.5)	392 (19.6)	50 (51.6)	64 (28.6)	36 (52.2)	37 (30.3)
No	245 (50.4)	1570 (78.7)	45 (46.4)	156 (69.6)	31 (44.9)	81 (66.4)

**Vertigo/dizziness**	χ^2^(2) = 121.07, *p* < 0.0001 (0.0001)		χ^2^(2) = 7.15, *p* = 0.028 (0.093)		χ^2^(2) = 3.78, *p* = 0.151 (0.382)	

Don’t know	32 (6.6)	63 (3.2)	4 (4.1)	10 (4.5)	1 (1.5)	6 (4.9)
Yes	196 (40.3)	380 (19)	42 (43.3)	63 (28.1)	26 (37.7)	32 (26.2)
No	258 (53.1)	1553 (77.8)	51 (52.6)	151 (67.4)	42 (60.9)	84 (68.9)

**Neck pain**	χ^2^(2) = 331.64, *p* < 0.0001 (0.0001)		χ^2^(2) = 42.92, *p* < 0.0001 (0.0005)		χ^2^(2) = 25.87, *p* < 0.0001 (0.002)	

Don’t know	9 (1.9)	27 (1.4)	1 (1)	6 (2.7)	0 (0)	2 (1.6)
Yes	318 (65.4)	460 (23.1)	70 (72.2)	73 (32.6)	50 (72.5)	42 (34.4)
No	159 (32.7)	1509 (75.6)	26 (26.8)	145 (64.7)	19 (27.5)	78 (63.9)

**Other pain syndromes**	χ^2^(2) = 128.77, *p* < 0.0001 (0.0001)		χ^2^(2) = 13.12, *p* = 0.001 (0.008)		χ^2^(2) = 7.7, *p* = 0.021 (0.128)	

Don’t know	17 (3.5)	23 (1.2)	1 (1)	3 (1.3)	2 (2.9)	3 (2.5)
Yes	199 (41)	373 (18.7)	51 (52.6)	70 (31.3)	34 (49.3)	36 (29.5)
No	270 (55.6)	1600 (80.2)	45 (46.4)	151 (67.4)	33 (47.8)	83 (68)

**Under psychatric treatment**	χ^2^(2) = 23.84, *p* < 0.0001 (0.0001)		χ^2^(2) = 3.83, *p* = 0.147 (0.316)		χ^2^(1) = 1.01, *p* = 0.316 (0.588)	

Don’t know	4 (0.8)	8 (0.4)	0 (0)	1 (0.5)	0 (0)	0 (0)
Yes	66 (13.6)	140 (7)	21 (21.7)	30 (13.4)	16 (23.2)	21 (17.2)
No	416 (85.6)	1848 (92.6)	76 (78.4)	193 (86.2)	53 (76.8)	101 (82.8)

**Diagnosed disease**	χ^2^(1) = 6.26, *p* = 0.012 (0.019)		χ^2^(1) = 0.74, *p* = 0.389 (0.648)		χ^2^(1) = 0.93, *p* = 0.335 (0.588)	

Yes	182 (37.5)	629 (31.5)	40 (41.2)	81 (36.2)	28 (40.6)	41 (33.6)
No	304 (62.6)	1367 (68.5)	57 (58.8)	143 (63.8)	41 (59.4)	81 (66.4)

*Pairwise comparisons using Pearson’s Chi-square test are reported. Percentages (%) displayed refer to column percentages. *p-*Values adjusted for multiple comparisons are shown between parenthesis and included variables from Table 3.*

## Discussion

The present study confirms previous findings from Vielsmeier et al. (2012) suggesting that tinnitus patients with TMJ complaints may constitute a separate subtype that could benefit from specific treatment interventions targeting the TMJ. Of major importance, we find that the proportion of TMJ complaints increases from 19% in the any tinnitus group to 36% in the severe group (as measured with the THI), strongly indicating that TMJ problems largely contribute to tinnitus severity. Many of the features analyzed in the present study showed a marked difference between tinnitus subjects with or without TMJ-complaints, similar to Vielsmeier et al. (2012). We confirm that a higher proportion of tinnitus subjects with TMJ complaints were female, younger, and could modulate tinnitus by head/jaw movements (Vielsmeier et al., 2012). The modulation of tinnitus by head/jaw movements has been previously reported (Pinchoff et al., 1998; Sanchez et al., 2002), and thus our work supports the hypothesis that somatic modulation may be associated with TMJ complaints in subjects with tinnitus (Levine, 1999). Somatic input from trigeminal afferents converge to the dorsal cochlear nucleus where granule cells contact fusiform cells that also integrate auditory input. It has been recently suggested that the plasticity of fusiform cells is perturbed during noise-induced tinnitus and that bimodal somatosensory and auditory stimulation of fusiform cells can reverse the plasticity to its original settings and provide relief from tinnitus in animals and humans (Marks et al., 2018).

While other studies found no difference between tinnitus patients with and without TMJ complaints in stress as an onset-related event (Vielsmeier et al., 2012; Ward et al., 2015), we reveal – using the same TSCHQ questionnaire – that a higher proportion of subjects in the group with TMJ complaints reported stress as a cause of their tinnitus. This is consistent with the greater level of anxiety and stress we have found in patients with TMJ complaints (Table 2). These emotional factors could not be evaluated in the study from Vielsmeier et al. (2012) since the questionnaire selection established by the TRI (Landgrebe et al., 2010) includes a questionnaire on depression [Beck Depression Index (BDI); Beck and Steer, 1984] but does not include measures of anxiety and stress. Indeed, stress questionnaires are seldom used in clinical practice. In the “any tinnitus” group, onset-related events were found to differ between TMJ and non-TMJ complaining groups. In contrast, this difference between any tinnitus and TMJ complaints tinnitus group was not found when only the severe tinnitus subjects were considered for each group. Since Vielsmeier et al. (2012) was studying a group of clinical patients, this could explain why they could not reveal any difference regarding onset-related events. This highlights the importance of including tinnitus subjects other than those in a clinical setting as it offers a better picture of the co-morbid factors, which would not be otherwise revealed.

Our findings also suggest that TMJ disorder, and potentially stress, may be a risk factor for the development of severe tinnitus. A large longitudinal study in Taiwan (7,585 TMJ disorders patients and 30,340 controls) supports this hypothesis showing a greater risk of developing tinnitus when diagnosed for TMJ disorders (adjusted HR = 2.75, 95% CI = 2.39–3.17) (Lee et al., 2016). Interestingly, previous studies have shown a strong correlation between bruxism and stress (Ahlberg et al., 2002, 2013), as well as between bruxism and tinnitus (Camparis et al., 2005; Fernandes et al., 2014). It has been suggested that distension of the TMJ capsule or antero-medial disk displacement could result in auriculotemporal nerve compression and facial pain symptoms (Cascone et al., 2010). Since a sustained clenching of the jaw can damage the articular disk (Commisso et al., 2014), it is reasonable to hypothesize that overloading the TMJ during stress periods may contribute to tinnitus. Studies in rodents have shown that auditory brainstem receives input from the trigeminal ganglion in rodents (Shore et al., 2000). However, there seems to be a large heterogeneity in the innervation of the TMJ capsule in humans (Davidson et al., 2003). To verify this, a longitudinal study could be performed looking at whether subjects with stress or/and sleep bruxism develop tinnitus over time.

The increased co-occurrence of TMJ complaints and tinnitus in women in the present study is indicative of a sex bias. These findings are consistent with greater tinnitus-related stress in women than in men (Seydel et al., 2013; Schlee et al., 2017a). Similarly, the psychological burden in women has also been recently evidenced by a greater association of tinnitus with suicide attempts, something which was not found in men (Lugo et al., 2019). However, sex-differences in TMJ complaints and tinnitus were also found in a Korean study (Won et al., 2013), but not in the United Kingdom (Ward et al., 2015). It is thus possible that the sex-specific impact of TMJ complaints on tinnitus may vary from one region to another, although this warrants further investigation. Indeed, little is known on the impact of sex on other factors related to tinnitus. The incorporation of sex as a biological variable (SABV) in tinnitus studies may help determining how sex impacts on the pathophysiology of tinnitus.

Our study also suggests that there is an increased occurrence of tinnitus in the family of those with TMJ complaints. We recently evidenced a genetic contribution to tinnitus in twins (Maas et al., 2017) and in adoptees (Cederroth et al., 2019b). In the latter study, we identified an association between adoptees and their biological parents, but not between adoptees and their adoptive parents, showing that the familial transmission of tinnitus is influenced by genetics and not by shared-environment. Previous genetic studies identified polymorphisms (i.e., those in the COMT gene) related to catecholamine metabolism and adrenergic receptors that may increase pain receptivity in subjects with chronic TMJ disorders (Diatchenko et al., 2005). Since tinnitus and chronic pain involve similar brain structures (Rauschecker et al., 2015), it is possible that the two conditions share common genetic mechanisms. However, as the genetic understanding of tinnitus is still in its infancy (Lopez-Escamez et al., 2016; Vona et al., 2017), large biobanking efforts will be needed to achieve a reliable understanding of the involvement of genetics in the co-occurrence of tinnitus and TMJ (Cederroth et al., 2017; Szczepek et al., 2018).

Tinnitus can affect an individual in many different ways (Hall et al., 2018), and there is no consensus measurement instrument for quantifying tinnitus symptom severity. In this study, we operationally defined severe tinnitus using scores derived from two popular self-report questionnaires; TFI and THI. This way, we could examine whether any observed differences between the TMJ and non-TMJ populations were measure-specific or robust regardless of how severe cases were defined. There is high convergent validity between the TFI and THI (*r* = 0.86, Meikle et al., 2012) indicating that they broadly measure the same construct, albeit with some small differences. In this dataset, the variables that differed regardless of the method used for defining severe cases were quality of life, influenced by head movement or touch, neck pain, or stress. Both TFI and THI assess concepts relevant to quality of life, and neither assess modulations by movement or touch, or associations with neck pain. Only the THI includes a question about exacerbation of tinnitus by stress. On this basis, it seems highly likely that the observations are robust, and not simply an artifact of the chosen methodology. Nonetheless, the proportion of subjects with severe tinnitus using the THI cut-off (7.7%) is smaller than that using the TFI cut-off (13%). Thus, a large proportion of subjects with clinically relevant tinnitus may not be identified using the arbitrary cut-offs from the THI, which have no true clinical meaning (Newman et al., 1998; McCombe et al., 2001). In contrast, the TFI cut-off we are using here is based on a revised grading using an anchor-based method tested on the United Kingdom population (Fackrell et al., 2017). These findings suggest that the inclusion of the TFI in regional guidelines of the Stockholm county (Idrizbegovic et al., 2011) could double the number of referrals to specialty care.

One limitation in the present study was that estimates of TMJ complaints were based on self-report rather than clinician assessment. Concerns have been raised on the reliance on self-reported symptoms and how accurate these are. There are also difficulties in finding agreement among clinical experts on how to diagnose somatosensory tinnitus (Michiels et al., 2018a). The single question from the TCSHQ – “*Do you suffer from Temporomandibular disorder?*” – is certainly not optimal from the point of view of questionnaire design and can be interpreted in a number of different ways, e.g., diagnosed by a doctor or not; uncertain response due to complex technical terminology. In addition, the “don’t know” option was not presented in the Vielsmeier study (Vielsmeier et al., 2012), which preempts from estimating the uncertainties related to the question itself. Instead, the Swedish version of the TSCHQ is reformulated in terms of jaw symptomatology, with only 6.3% of “don’t know” answers. Further studies would be needed to relate tinnitus severity with objective measures of TMJ disorders such as MRI or CT-scan (Scrivani et al., 2008), in addition to being diagnosed by a doctor (e.g., through checking for the presence of a clicking sound) and including TMJ disorders laterality in the analysis prior performing more costly evaluations of treatment interventions. Consistent with the lack of objective measures for both TMJ disorders and tinnitus, high quality studies evaluating the effect of TMJ disorders treatment on tinnitus are missing (Skog et al., 2019). A recent systematic review by Michiels et al. (2019) reveals that the combination of splint therapy and exercise treatment, the so-called conservative treatment, is the best treatment approach as several studies show a decrease in tinnitus severity. However, the overall evidence shows low-quality and argues in favor of larger well-designed studies, such as the one ongoing by Michiels et al. (2018b).

Identification of distinct tinnitus subtypes will be critical for successful therapeutic development. Our analysis confirmed that TMJ problems are closely tied to socioeconomic, phenotypic, and psychological features of individuals with tinnitus. Our study supports the notion that tinnitus subjects with TMJ complaints could constitute a specific subtype. From a clinical trial perspective, this subgroup of tinnitus patients should therefore be carefully considered in exclusion/inclusion criteria as their response to treatments could dramatically differ from other tinnitus patient groups.

## Data Availability

The datasets analyzed in this manuscript are not publicly available. Requests to access the datasets should be directed to the corresponding author.

## Author Contributions

CC, DH, and BC designed the study. CC and NE developed the websurvey, coordinated the recruitment of subjects, and collected the data. NE extracted and processed the STOP data. NE, EdG, JB, and ElG analyzed the data. AL, GM, MB, MT, and JW helped to develop the scientific arguments and contributed to the data interpretation. All authors contributed to the manuscript and approved the final version.

## Conflict of Interest Statement

GM and JW are employees of Decibel Therapeutics. The remaining authors declare that the research was conducted in the absence of any commercial or financial relationships that could be construed as a potential conflict of interest.

## Footnotes

1 https://stop.ki.se

2 https://www.tinnitusresearch.net/
